# EP4 receptor stimulation in combination with core decompression therapy enhanced bone regeneration in a canine model of osteonecrosis of femoral head

**DOI:** 10.3389/fbioe.2025.1622918

**Published:** 2025-08-14

**Authors:** Kyoko Hirano, Takaki Miyagawa, Masahiro Nishida, Haruhiko Akiyama

**Affiliations:** ^1^ Department of Orthopaedic Surgery, Gifu University Graduate School of Medicine, Gifu, Japan; ^2^ Laboratory for Pharmacology, Pharmaceutical Research Center, Asahi Kasei Pharma Corporation, Shizuoka, Japan; ^3^ Center for One Medicine Innovative Translational Research (COMIT), Gifu University, Gifu, Japan

**Keywords:** EP4, prostaglandin, bone regeneration, osteonecrosis, femoral head, dog, animal model

## Abstract

Core decompression (CD) is a minimally invasive procedure widely used to treat early-stage osteonecrosis of the femoral head (ONFH). However, CD alone often yields suboptimal outcomes in promoting bone regeneration in necrotic lesions, highlighting the need for novel therapeutic approaches. In this study, we evaluated the combined effects of CD surgery and local administration of AKDS001, a small-molecule EP4 receptor agonist, in a canine ONFH model. AKDS001 was incorporated into biocompatible, biodegradable polylactic-coglycolic acid microspheres (AKDS001 MSs) for sustained local drug release. The bone-regenerative effects of local administration of AKDS001 MSs combined with CD surgery were evaluated in intact canines or a canine ONFH model, induced by ethanol injection into the femoral head. Safety and local tolerability of the therapy was also investigated in the model. AKDS001 MSs enhanced bone formation in intact dog femurs compared to CD only or MSs without AKDS001. In the ONFH model, CD alone resulted in limited bone repair at 12 weeks postsurgery. In contrast, compared with CD alone, the combination of AKDS001 MSs and CD dose-dependently increased the bone volume, bone mineral density, and tissue mineral density in the CD tunnel. Histological analyses further revealed significant amelioration of the necrotic lesions. Importantly, no systemic or local adverse effects were observed. In conclusion, local administration of AKDS001 MSs combined with CD surgery significantly enhanced bone regeneration in necrotic lesions in a canine ONFH model, demonstrating both efficacy and favorable safety with local tolerability.

## 1 Introduction

Osteonecrosis of the femoral head (ONFH) is a well-recognized, debilitating condition characterized by avascular necrosis of the femoral head, leading to its collapse (Mont et al., 2020; Hines et al., 2021). ONFH incidence is commonly associated with systemic steroid use, habitual alcohol intake, and smoking. The primary goal of ONFH treatment is to prevent femoral head collapse in the early stage of the disease. In the United States, ONFH is the underlying diagnosis in 10% of all total hip arthroplasty (THA) cases (Moya-Angeler et al., 2015). Although the prosthetic implants used in THA can last approximately 25 years (Evans et al., 2019), implant longevity and the subsequent need for revision surgery remain outstanding problems among ONFH patients (Petek et al., 2019), who are mainly young and active adults. Therefore, there is a high medical need for a novel therapeutic approach to delay or prevent THA.

Core decompression (CD) is a hip-preserving surgical procedure that involves drilling and removing a cylindrical core from the necrotic lesion (Moya-Angeler et al., 2015). CD is well recognized for its ability to temporarily reduce intramedullary pressure, providing short-term clinical improvement and partial or complete pain relief in ONFH patients. However, its effectiveness in stimulating osteogenic cells and osteoinductive signaling proteins, such as growth factors and cytokines, remains limited (Ando et al., 2021). Therefore, new therapeutic approaches to administer growth factors (Sun et al., 2014; Shi et al., 2017; Lieb et al., 2004; Papanagiotou et al., 2014; Kuroda et al., 2021) and/or cells (Hernigou et al., 2018; Tomaru et al., 2022; Maruyama et al., 2019) locally at the same time as CD surgery to enhance bone regeneration have been explored extensively in recent decades; however, these approaches have not yet been approved. We recently demonstrated that the clinical application of a controlled-release recombinant human fibroblast growth factor (rhFGF)-2-loaded gelatin hydrogel promoted bone regeneration in necrotic areas in patients with precollapse ONFH (Kuroda et al., 2021). Despite these advancements, small-molecule drug approaches offer a compelling alternative considering their scalability, costs for manufacturing and quality control, and ease of clinical application (Presciutti and Boden, 2018; Laurencin et al., 2014; Beck et al., 2022).

AKDS001, a highly potent and selective small-molecule agonist of the EP4 receptor, represents a promising candidate for ONFH therapy. The EP4 receptor is a critical mediator of prostaglandin E2 (PGE2)-induced osteogenesis, promoting bone formation by osteoblasts (Jee and Ma, 1997; Ke et al., 1998; Weinreb et al., 1999; Machwate et al., 2001; Yoshida et al., 2002; Norel et al., 2020). While preclinical studies have highlighted the therapeutic potential of EP4 receptor agonists for osteoporosis and bone repair, their preclinical evaluation in nonrodent animals remains limited, and clinical trials have yet to be performed. We developed a novel formulation with polylactic-co-glycolic acid (PLGA) microsphere (MS) technology to achieve the local sustained release of AKDS001 (Ukon et al., 2022). Furthermore, AKDS001 MSs injection in combination with human bone xenograft transplantation significantly increased osteogenic activity in athymic nude rats in a dose-dependent manner, which was driven by increased osteoblast function.

In this study, we evaluated the efficacy of AKDS001 MSs in a canine model of ONFH, in which osteonecrosis was induced by a single intraosseous injection of ethanol. This well-established method replicates the pathological features of early-stage ONFH (Wang et al., 2013; Manggold et al., 2002). Dogs are widely used in preclinical drug development as anatomically relevant non-rodent models and exhibit EP4 receptor-mediated responses to AKDS001 that are comparable to those observed in humans (Ukon et al., 2022). This model enabled us to assess the potential of AKDS001 to promote bone regeneration within necrotic bone tissue under clinically relevant conditions.

## 2 Materials and methods

### 2.1 Ethics statement

All experiments involving human cells and tissues were approved on 22 June 2020, by the institutional review boards of Gifu University (2019-284) and Asahi Kasei Pharma Corporation (20003) in accordance with the World Medical Association Declaration of Helsinki. Human femoral head tissues were collected from donors who had undergone hip replacement surgery for steroid-associated ONFH or hip fracture. Informed consent was obtained from all donors for the use of their tissues in the study. The protocols for the animal studies were approved by the institutional animal ethics committees (19-148, 19-266, 20-191, 21-201, 21-203) and conducted in compliance with established guidelines for the care and use of experimental animals.

### 2.2 Chemicals

The selective EP4 agonist, AKDS001 (Asahi Kasei Pharma, Tokyo, Japan), was incorporated into biocompatible and biodegradable polylactic coglycolic acid (PLGA) microspheres (MSs) for sustained drug release (Ukon et al., 2022). The ratio of polylactic acid to polyglycolic acid was 1:1. PLGA MSs with AKDS001 (AKDS001 MSs) and without AKDS001 (Blank MSs) were used for the following animal experiments. AKDS001 in PLGA MSs was released sustainedly approximately for 6 weeks.

### 2.3 *In vitro* study

Human mesenchymal stem cells (MSCs) and femoral head tissues were obtained from patients aged 27–94 years who underwent hip replacement surgery due to either idiopathic ONFH associated with corticosteroid use or age-related hip fracture. ONFH samples were included to examine whether EP4 receptor expression is altered in osteonecrotic bone, particularly in the context of ONFH associated with steroid use, which has been reported to impair MSC function (Houdek et al., 2016). Hip fracture samples were used as a non-ONFH reference, representing typical clinical controls for degenerative bone without osteonecrosis.

Each femoral head tissue sample was divided into two parts. One-half was fixed in 10% neutral buffered formalin for histological analysis, while the other half was stored in MSC growth medium (Lonza K.K., Kanagawa, Japan) to isolate bone marrow-derived MSCs. For histological analyses, the remaining formalin-fixed tissue samples were trimmed, sliced into 5 mm thick sections, and then decalcified in ethylenediaminetetraacetic acid solution (100 mg/mL, pH 7.2). Paraffin-embedded blocks and sections were prepared at Kureha Special Laboratory Co., Ltd. (Tokyo, Japan). Immunohistochemical staining for EP4 was performed using a rabbit anti-EP4 antibody (1.0 mg/mL, NLS3890; Novus Biologicals, Centennial, CO, United States) or rabbit IgG (I-1000; Vector Laboratories, Newark, CA, United States) as a negative control. The sections were deparaffinized, incubated in 0.5% Tween 20/PBS for 6 h, blocked with 1.5% normal goat serum/0.05% Tween/PBS for 1 h, and treated with primary antibodies overnight at 4°C. The detection of rabbit IgG primary antibodies was performed using the VECTASTAIN ABC-HRG rabbit IgG kit (Vector Laboratories).

MSCs were isolated using a modified version of a previously reported protocol (Vemuri et al., 2011; Grisendi et al., 2010). Briefly, bone tissue samples were rinsed with Hank’s balanced salt solution (HBSS) to obtain bone marrow suspensions. The suspension was collected and gently overlaid onto Ficoll-Paque PREMIUM 1.073 (Cytiva, Tokyo, Japan) in a plastic tube, followed by centrifugation at 400 × *g* for 30 min. The buffy coat at the Ficoll–HBSS interface was collected, diluted with HBSS, and centrifuged again. The cells were resuspended in MSC growth medium and incubated in plastic plates at 37°C with 5% carbon dioxide for more than 24 h to allow adherent cells to attach. Commercially sourced human MSCs from PromoCell (Heidelberg, Germany) and Lonza K.K served as healthy controls. MSCs at passage 4 were treated with AKDS001 (Asahi Kasei Pharma Corporation) or PGE2, and intercellular cAMP production was measured as previously described (Machwate et al., 2001; Ukon et al., 2022). The half-maximal effective concentration (EC_50_) values for each donor were calculated using GraphPad Prism 7 (MDF Co., Ltd., Tokyo, Japan).

### 2.4 Intact femur dog study

Prior to the model study, effects on bone formation of AKDS001 MSs in the dog femoral head was preliminarily investigated in the femurs without osteonecrosis. A total of 18 female beagle dogs aged 12–13 months (Kitayama Labes Co., Ltd., Nagano, Japan) were used in this study. AKDS001 MSs and blank MSs were obtained from Asahi Kasei Pharma Corporation. The bilateral femurs of 12 dogs (24 hips) were subjected to either CD alone (drilling only; n = 4) or CD in combination with 3 or 10 µg/site of AKDS001 MSs (n = 4 for each dose) or equivalent doses of blank MSs (n = 4 for the low dose and n = 8 for the high dose) according to the assignment (Supplementary Table S1). The other six animals (12 hips) received the intraarticular injections of AKDS001 MSs or Blank MSs. The minimum sample sizes were chosen to detect the tendency of bone formation. *In vivo* computed tomography (CT) imaging (R_mCT3 AX, Rigaku, Tokyo, Japan) was performed under general anaesthesia with isoflurane before surgery, immediately after surgery, and every 2 weeks thereafter. The animals were humanely euthanized 6 weeks after drug administration. The femurs subjected to CD surgery were scanned using the Scan Xmate-RB090SS150 device, and the bone volume to total volume (BV/TV) ratio in the CD tunnel was analysed using TRI/3D-BON-FCS. The ROI for the CD tunnel was defined as a 2 mm-diameter cylinder with a length covering the entire CD tunnel. Following CT imaging, the proximal regions of the femurs were decalcified, embedded in paraffin, and prepared for histological analysis. Hematoxylin and eosin (HE)-stained coronal sections were obtained for detailed histological examination.

### 2.5 Surgery for intact femur dogs

All the animals completed a 5-day acclimation period and were then determined to be healthy and fit for use prior to the start of the study. During the study, information about the treatment groups was masked from the surgeons to eliminate bias. Under general anaesthesia induced by intravenous injection of 20 mg/kg thiopental sodium and maintained by inhalation of 1%–4% isoflurane (Mylan, Tokyo, Japan), a 2-mm-diameter Kirschner wire was inserted into the femoral head from the distal end of the greater trochanter under aseptic conditions, followed by an injection needle for the delivery of AKDS001 MSs or blank MSs suspensions. For intra-articular injections, the femoral head was punctured with a 2-mm-diameter Kirschner wire to access the intra-articular space. Thereafter, the surgical incisions were closed in multiple layers in accordance with standard surgical techniques. The animals were treated with cefotaxime (0.05 g/kg, i.m., twice/day) and butorphanol tartrate (0.5 mg/kg, i.m., twice/day) on the day of and 3 days after drug injection. The animals were individually housed to avoid contact with one another during recovery.

### 2.6 ONFH model study design

**FIGURE 1:**
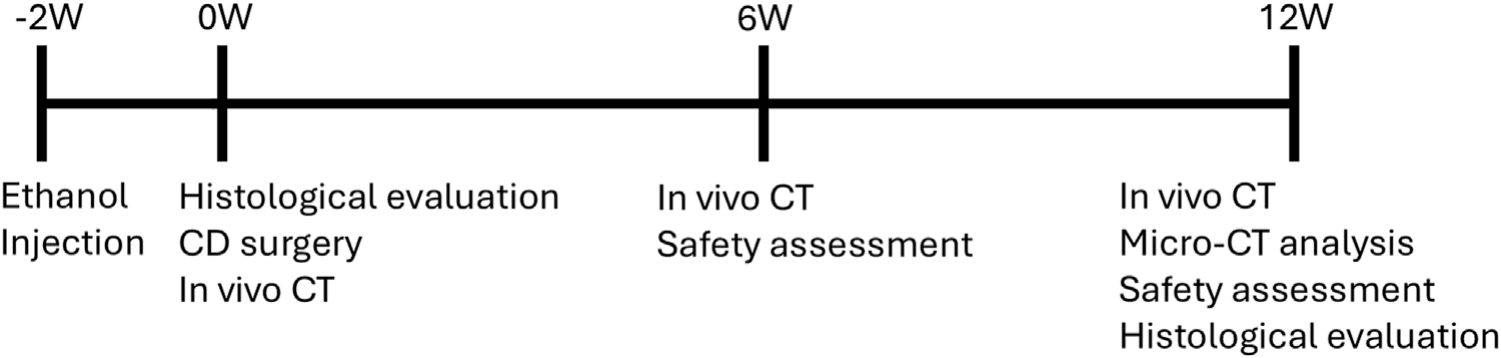
Timeline of the ONFH model study. W, week; CD, core decompression.

Female adult beagle dogs aged 9–14 months (Kitayama Labes Co., Ltd., Yamaguchi, Japan) were used in this study. The observation period was set at 12 weeks after dosing (Figure 1), which is two times longer than the intact femur study. To obtain baseline data for the model, four animals received ethanol injection into the femoral head for model development and were euthanized at 2 weeks for histological assessment. To evaluate the efficacy of AKDS001 MSs, forty animals that received ethanol injection into the left femur were divided into 4 groups (n = 10 per group) after 2 weeks. The control group underwent CD alone, whereas the other three groups received CD in combination with AKDS001 MSs at doses of 1, 3, and 10 µg per site. The other 10 animals (n = 5 per group) received similar procedures and were treated with CD alone or with 3 µg of AKDS001 MSs per site for qualitative histological analysis. Those doses and sample sizes were determined based on the results of the intact femur dog study, and the CD only group was used as a control because there was no clear difference detected between the blank MS and CD only groups in the intact femur dog study. *In vivo* CT images under general anesthesia with isoflurane were acquired within 1 week of the CD procedure (0 weeks) and at 6 and 12 weeks postsurgery. One animal in the 1 µg per site group and one in the 3 µg per site group were excluded from the study because of infection associated with the bone wax applied during the surgery. All the animals were sacrificed at 12 weeks after CD surgery, and the femurs were extracted for micro-CT imaging and histological assessment. For safety assessment, an additional 12 animals underwent bilateral ethanol injections and CD surgery. Two groups were tested: one group receiving a dose 10 times higher than the effective dose (100 µg/site of AKDS001 MS, 200 µg/body) and one group treated with CD only (n = 6 per group). Half of the animals in each group (3 animals per group) were euthanized at 6 weeks post-treatment, and the remaining animals were euthanized at 12 weeks.

### 2.7 Development of the ONFH dog model

All the animals completed at least a 5-day acclimation period and were then determined to be healthy and fit for use prior to the procedure. To establish an ONFH model, dogs received ethanol injection into the femoral head, as previously reported (Wang et al., 2013; Manggold et al., 2002). Briefly, the animals were anaesthetized with 80 mg of propofol and isoflurane for aseptic surgery. An incision in the skin covering the distal tip of the greater trochanter was created. The tensor fascia was incised, and the soft tissues were detached from the distal side of the trochanter. A 1.2-mm needle was inserted into the left femoral head through the trochanter area under fluoroscopic guidance, following the same procedure as for CD surgery. Pure ethanol (>99%; FUJIFILM Wako Pure Chemical Corporation, Osaka, Japan) was then injected through the needle using a syringe pump at a rate of 0.2 mL/min for 5 min. An anchor wire was implanted into the puncture hole used for ethanol injection. The fascia, subcutaneous tissues, and skin were sutured to close the incisions. Postoperative care included intramuscular administration of buprenorphine (0.05 mg/body, twice/day) on the day of surgery and for 5 days afterwords, along with cefotaxime (0.5 g/body) on the day of surgery and for 3 days post-surgery.

### 2.8 CD surgery for the model animals

CD surgery under general anesthesia was performed by surgeons blinded to the treatment groups at 2 weeks after ethanol injection. The procedures used for surgery and postoperative care were similar to those used for model development. CD tunnels were created with the aid of fluoroscopic guidance, and the anchor wire was implanted into the puncture hole used for ethanol injection. A 3-mm-diameter cannulated drill was inserted into the femoral head through the distal end of the greater trochanter, followed by an injection needle to administer the MS suspension. Immediately after the CD procedure, the puncture hole was covered with bone wax.

### 2.9 Quantitative analysis of CT images

At 12 weeks post-treatment, the animals were euthanized, and the bilateral femurs were harvested and fixed in 10% neutral buffered formalin. Micro-CT imaging of the samples was performed with nanoScan SdC (Mediso, Budapest, Hungary) and VivoQuant (Invicro, Needham, MA, United States) at Invicro, LLC to quantitatively analyze the extent of bone repair in the CD tunnels. The regions of interest (ROIs) were identified on the left femur and the corresponding anatomical positions on the right contralateral femur to measure the bone mineral density (BMD), the BV/TV, and the tissue mineral density (TMD). The entire CD tunnel was designated ROI-1. A subregion corresponding to the tip of the CD tunnel (6.25 mm from the tip, located within the femoral head) was separated from ROI-1 and defined as ROI-2. The CT parameters of the left treated femur were compared with those of the right contralateral intact femur to assess the effects of the treatments.

### 2.10 Histological evaluation

To observe the baseline of the model, proximal femurs at 2 weeks after ethanol injection was fixed in 10% neutral buffered formalin and embedded in paraffin blocks. A single section including the drill hole for ethanol injection was obtained from each femur and evaluated by a pathologist. For histological assessment at 12 weeks, the proximal femurs were embedded in methyl methacrylate at Histion, LLC (Everett, WA, United States), and two thin sections were prepared along a plane passing through the approximate center of the CD tunnel. One section from each pair was stained with toluidine blue, while the other was stained with HE. A pathologist, blinded to the treatment groups, performed histological scoring using a scoring system (Table 1; Supplementary Table S2) established before the assessment, with modifications to a published method (MacBarb et al., 2017). For quantitative histological analysis, decalcified paraffin-embedded sections were prepared. The bone area, the number of osteocyte-occupied lacunae (normal lacunae), and the number of empty lacunae were automatically measured or counted using Visiopharm software (Visiopharm A/S, Hørsholm, Denmark) in the femoral head outside the CD tunnel. The ROI for the femoral head was placed within 15 mm of the femoral neck from the proximal end of the femur. The bone area was identified *via* eosin staining. In the bone area, white particles were counted as empty lacunae, whereas hematoxylin-stained particles were counted as normal lacunae.

**TABLE 1:** Scoring criteria for histological assessments.

Necrosis	Score	Fibrosis	Score
Extensive: necrosis throughout the treatment area and beyond	4	Present in >25% of the area within and around the treatment area	4
Moderate amount: present in all or a large portion of the treatment area and/or beyond	3	Present in 15%–25% of the area within and around the treatment area	3
Small amount: present in a moderate portion of the treatment area and/or beyond	2	Present in 5%–15% of the area within and around the treatment area	2
Very small amount: present in a small portion of the treatment area and/or beyond	1	Present in 1%–5% of the area within and around the treatment area	1
None observed	0	Present in <1% of the area within and around the treatment area	0

### 2.11 Safety assessment

Safety evaluations, including ophthalmology, electrocardiography, hematology, clinical chemistry, and urinalysis, were conducted at 6 weeks and 12 weeks after CD surgery. Blood sampling for toxicokinetics was performed at pre-dosing, 1, 2, 4, and 24 h postdosing and 8, 15, 22, 29, 33, 36, 43 and 50 days postdosing. Approximately 1.8 mL/point of blood was collected and used to measure the plasma concentration of AKDS001. The animals were euthanized at 6 or 12 weeks after dosing for gross pathology, and the organs, including the hearts, thymuses, spleens, bronchi, lungs, submandibular glands, livers, gallbladders, pituitaries, thyroids, parathyroids, adrenals, ovaries, uteruses, and brains, were weighed. The bilateral hip joints, including the proximal femurs, were extracted from all the animals. HE staining of the hip joint was used to evaluate local tolerability. All the assessments were performed in compliance with GLP Standards for nonclinical safety studies of drugs (ministry of health and welfare ordinance No. 21, Mar. 26, 1997, Japan) and partial revision of the guidelines for repeated-dose toxicity studies (notification No. 655, Apr. 5, 1999, ministry of health and welfare, Japan) at SNBL INA Ltd. (Tokyo, Japan).

### 2.12 Statistical analysis

Statistical analysis of CT data and qualitative histological assessments was conducted using Prism 7 (MDF Co., Ltd., Tokyo, Japan). Differences from the control group were evaluated using Dunnett’s multiple-comparison test for CT analysis and t-test for qualitative histological assessments. The statistical analysis for the histological assessment was performed with Statistica, version 14 (TIBCO Software Inc., Palo Alto, CA, United States). Differences between groups were determined using the Kruskal–Wallis multiple-comparisons test. A p value of ≤0.05.

## 3 Results

### 3.1 *In vitro* study

**FIGURE 2:**
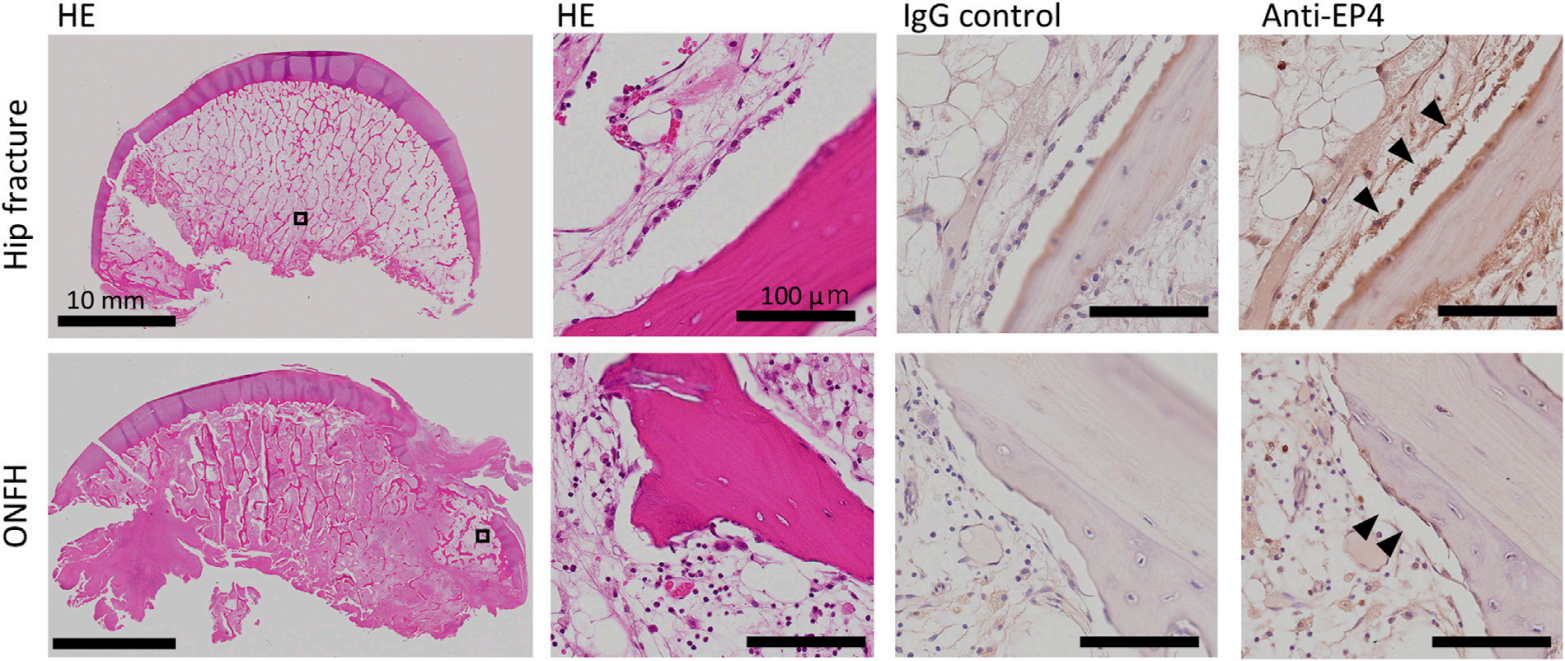
HE staining and immunohistochemistry of human femoral heads from patients with hip fracture (upper panels, 66 years old, female) and ONFH (lower panels, 73 years old, female). The black boxes are shown at higher magnification. Arrowheads indicate EP4-positive lining cells. HE, hematoxylin–eosin; ONFH, osteonecrosis of the femoral head.

In the immunohistochemical analysis, EP4 expression was observed in the cells lining the surface of appositional bone with an osteoblast-like morphology in the femoral heads derived from patients with ONFH as well as patients with hip fracture (Figure 2). This finding indicates the presence of EP4 receptors, which aretargets of AKDS001,even in necrotic bone tissue. In human bone marrow-derived MSCs, the half-maximal effective concentration (EC_50_) values of AKDS0001 for increasing intracellular cAMP were similar between the two types of donors (patients with hip fracture and patients with ONFH) and the healthy controls (Supplementary Table S3), suggesting that the EP4 agonistic activity of AKDS001 was not decreased in ONFH patients with a history of steroid use.

### 3.2 Effects of AKDS001 MS on the intact dog femurs

**FIGURE 3:**
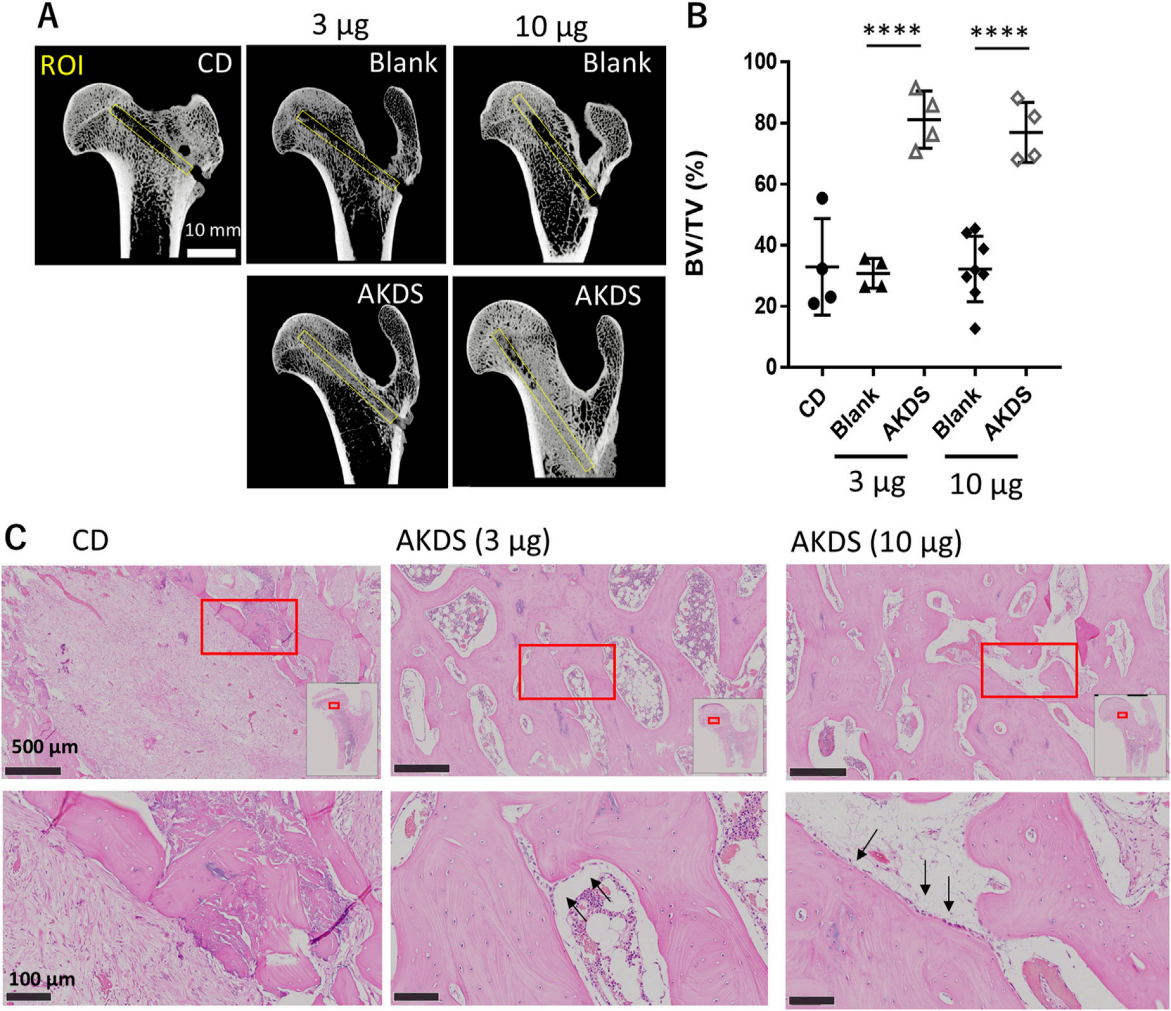
**(A)** Micro-CTimages of extracted femurs. Yellow boxes indicate the ROIs for bone volume measurement placed on the CD tunnel. **(B)** Results of bone volume measurement for the ROIs. Plots indicate individual values. The bars indicate the means ± standard deviations. **(C)** Histology of the CD tunnels. Red boxes are shown at higher magnification. The white box in the upper panel shows the map of the proximal femurs. The black arrows indicate lining cells on the bone surface. CD, core decompression; blank, blank microspheres; AKDS, AKDS001 microspheres; ROI, region of interest; BV/TV, bone volume/total volume. ****p < 0.0001.

Six weeks after CD surgery, the bone volume in the CD tunnel remained relatively low in the CD (n = 4) and blank MS groups (n = 4 for the low-dose group and n = 8 for the high-dose group). Quantitative analysis of the CD tunnels with micro-CT imaging revealed that AKDS001 MSs (n = 4 for 3 and 10 µg/site, respectively) significantly increased the bone volume compared with blank MS, whereas the bone volumes in the blank MS groups were similar to that in the CD group (Figures 3A,B). *In vivo* CT images revealed new bone formation starting at approximately 4 weeks after dosing (Supplementary Figure S1). In the histological sections, the CD control group presented fibrotic tissues and bone debris within the CD tunnels, whereas the AKDS001-treated groups presented better bone filling in the CD tunnels than did the CD group. (Figure 3C). Lining cells were observed on the surface of the newly formed bone in the CD tunnels in the AKDS001-treated groups. In contrast, intra-articular injection of AKDS001 MS resulted in no clear bone formation in the hip joint space, as shown by *in vivo* micro-CT imaging and gross observation at 6 weeks after injection (Supplementary Figure S2).

### 3.3 Establishment of the ONFH dog model

**FIGURE 4:**
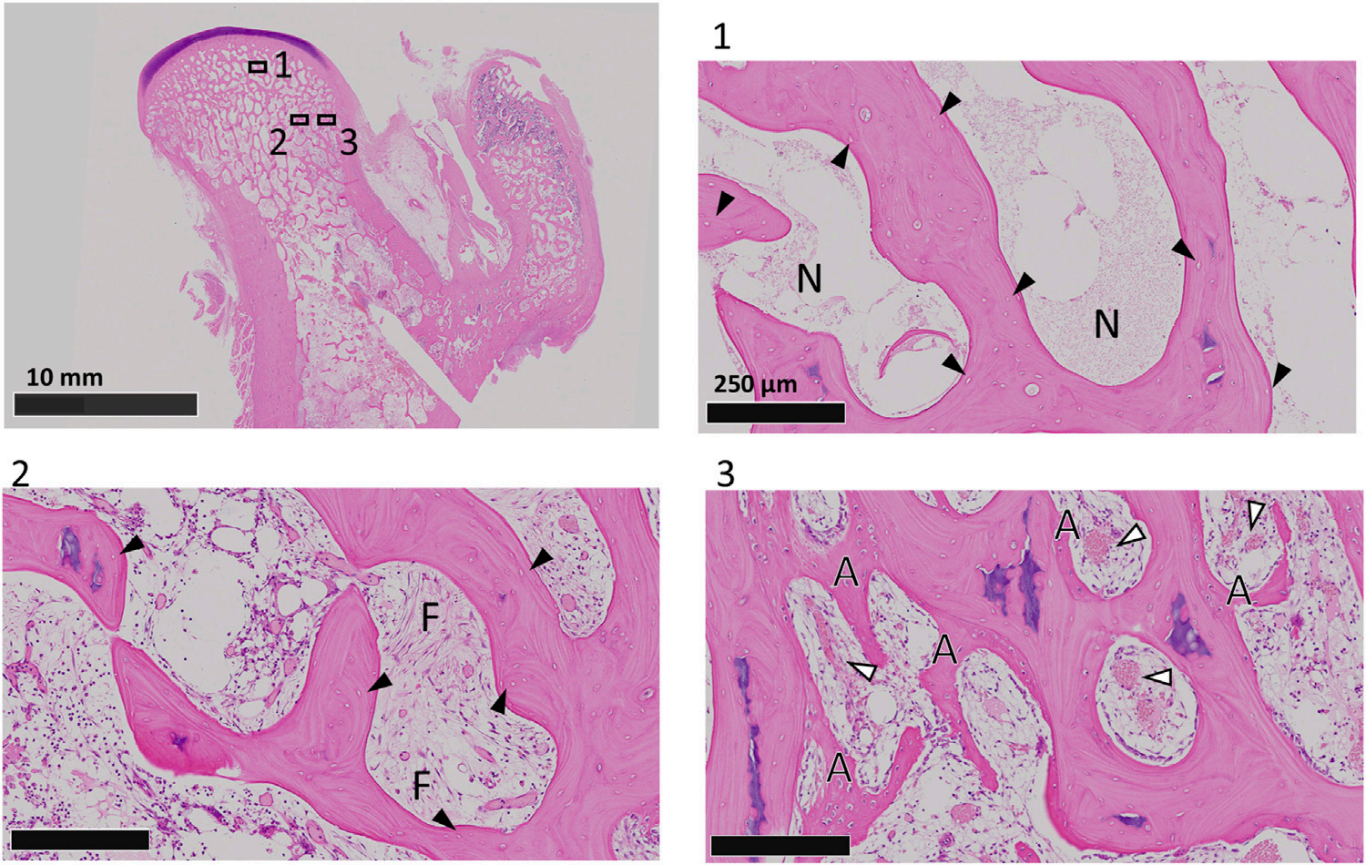
HE staining of coronal sections of dog femoral heads at 2 weeks after ethanol injection. Boxed regions are shown at higher magnification. Black arrowheads indicate empty lacunae. The white arrowheads indicate blood vessels. N, necrotic bone marrow; F, fibrotic bone marrow; A, appositional bone.

Two weeks after receiving a direct intraosseous injection of pure ethanol into the femoral head, all the animals presented necrotic bone tissues in the femoral head, including necrotic bone with empty lacunae and necrotic bone marrow surrounded by necrotic or fibrotic bone marrow (Figure 4). These histological changes were similar to those reported in previous studies (Wang et al., 2013) and the pathological features reported in clinical patients with ONFH (Cui et al., 2021). On the other hand, in the peripheral region of the ethanol injection route, bone apposition and vascularization were observed. These findings indicate that necrosis was established within 2 weeks after ethanol injection and was localized specifically to the injection site of ethanol, where we performed CD surgery.

### 3.4 Effects of AKDS001 MS in the ONFH dog model

**FIGURE 5:**
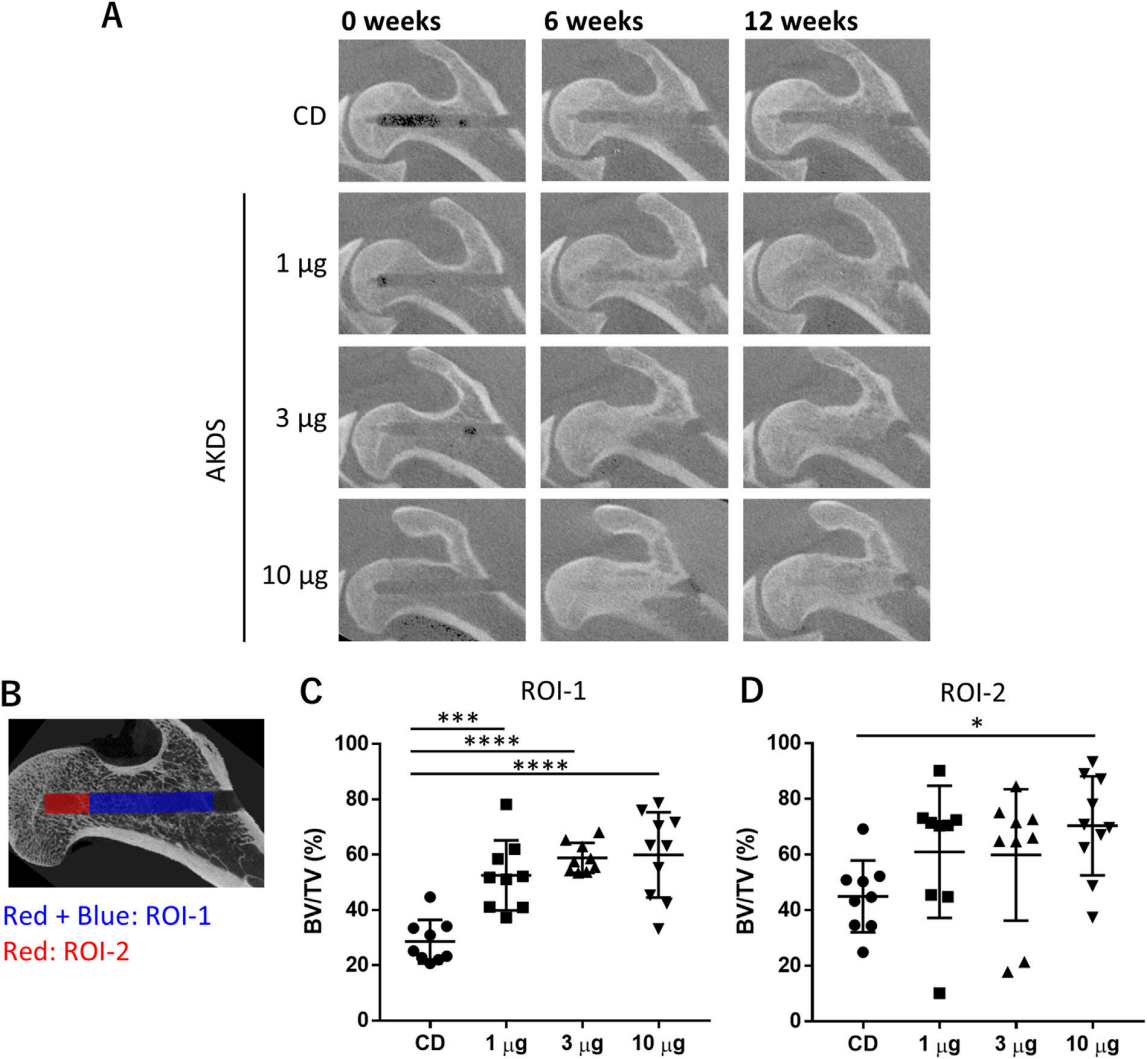
**(A)** Representative *in vivo* computed tomography images of dog proximal femurs. **(B)** ROIs for micro-CT analysis. The red- and blue-colored regions indicate ROI-1, and the red-colored region indicates ROI-2. **(C, D)** Results of bone volume measurement in ROI-1 and ROI-2, respectively. CD, core decompression; AKDS, AKDS001 microspheres; BV/TV, bone volume/total volume; ROI, region of interest; *p < 0.05, **p < 0.01, ***p < 0.001, ****p < 0.0001. Error bar: standard deviation.

*In vivo* CT images revealed that the CD tunnel in the CD group was apparent during the study period (Figure 5A). The edge of the CD tunnels was more ambiguous in the AKDS001 MS-treated groups than in the CD-only group. New bone formation was evident in the AKDS001 MS-treated groups at 6 and 12 weeks after dosing.

According to the micro-CT analysis of the entire CD tunnels (ROI-1), as shown in Figure 5B, the BV/TVs in the CDs of the treated femurs were significantly greater in the AKDS001 MS-treated groups at all the tested doses (n = 9 for 1 and 3 µg/site, respectively; n = 10 for 10 µg/site) than in the control group (n = 9) (Figure 5C). Additionally, in ROI-2, which was located at the tip of the CD tunnel as shown in Figure 5B, the bone volume was increased by AKDS001 treatment in a dose-dependent manner, and the difference between the high-dose group and the control group was statistically significant (Figure 5D). The changes in the BMD and tissue mineral density tended to be similar to those in the BV/TV (Supplementary Tables S4 and S5), suggesting that the increased bone tissue underwent more effective mineralization in the AKDS001 MS-treated groups than in the control group.

The increases in BV/TV and BMD were comparable to or greater in the AKDS001 MS-treated groups than in the intact femur group. In addition, no significant differences among the groups were noted in the contralateral femurs, which were left intact. Thus, AKDS001 MSs facilitated bone regeneration specifically at the injection site without any systemic effects, including new bone formation outside the femoral head.

**FIGURE 6:**
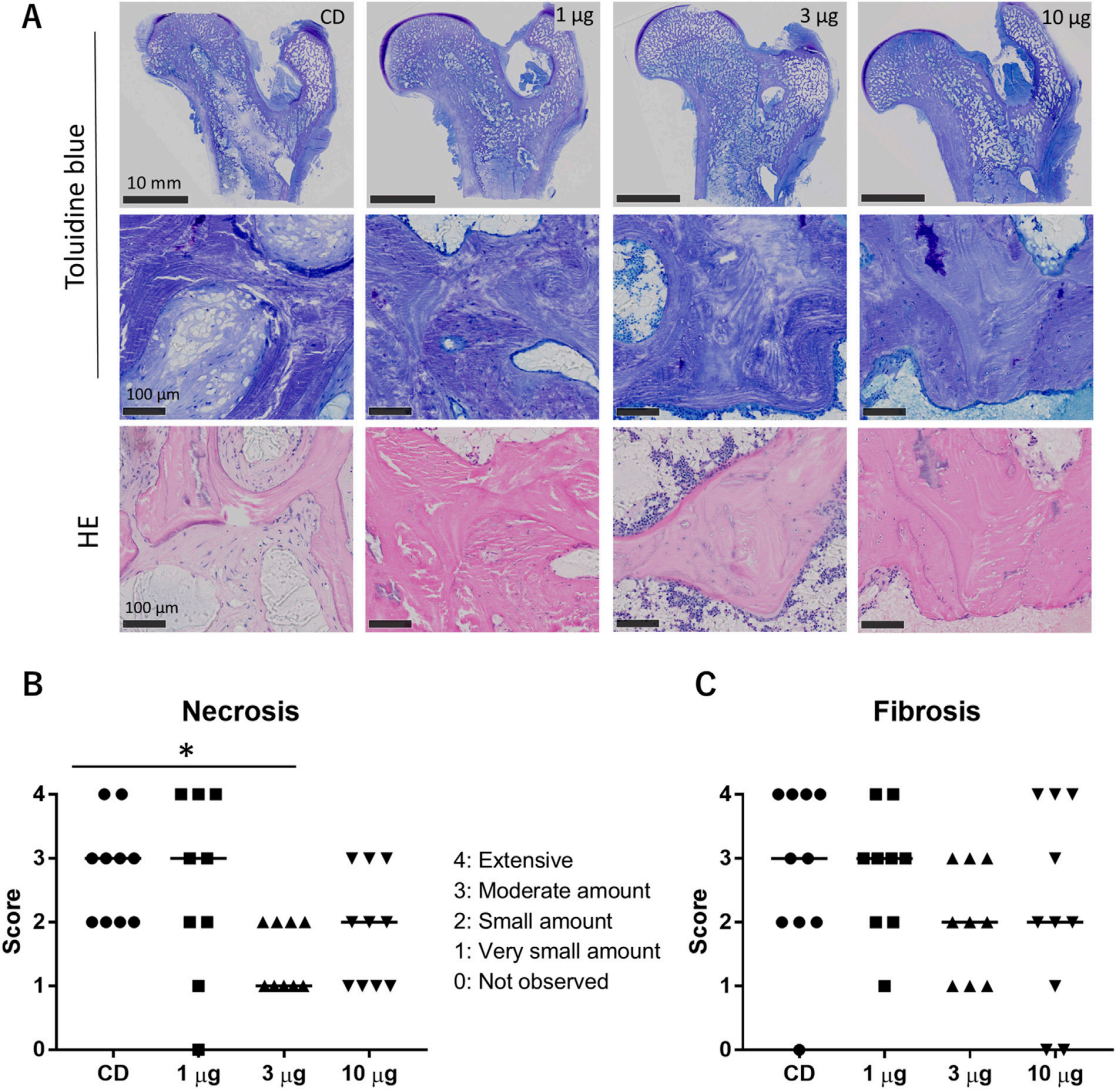
**(A)** Histology of toluidine blue-stained sections (upper and middle panels) and HE-stained sections (lower panels). **(B, C)** Results of the histological assessment of necrosis **(B)** and fibrosis **(C)**. CD, core decompression; AKDS, AKDS001 microspheres; HE, hematoxylin–eosin. *p < 0.05. Bar: median score.

**FIGURE 7:**
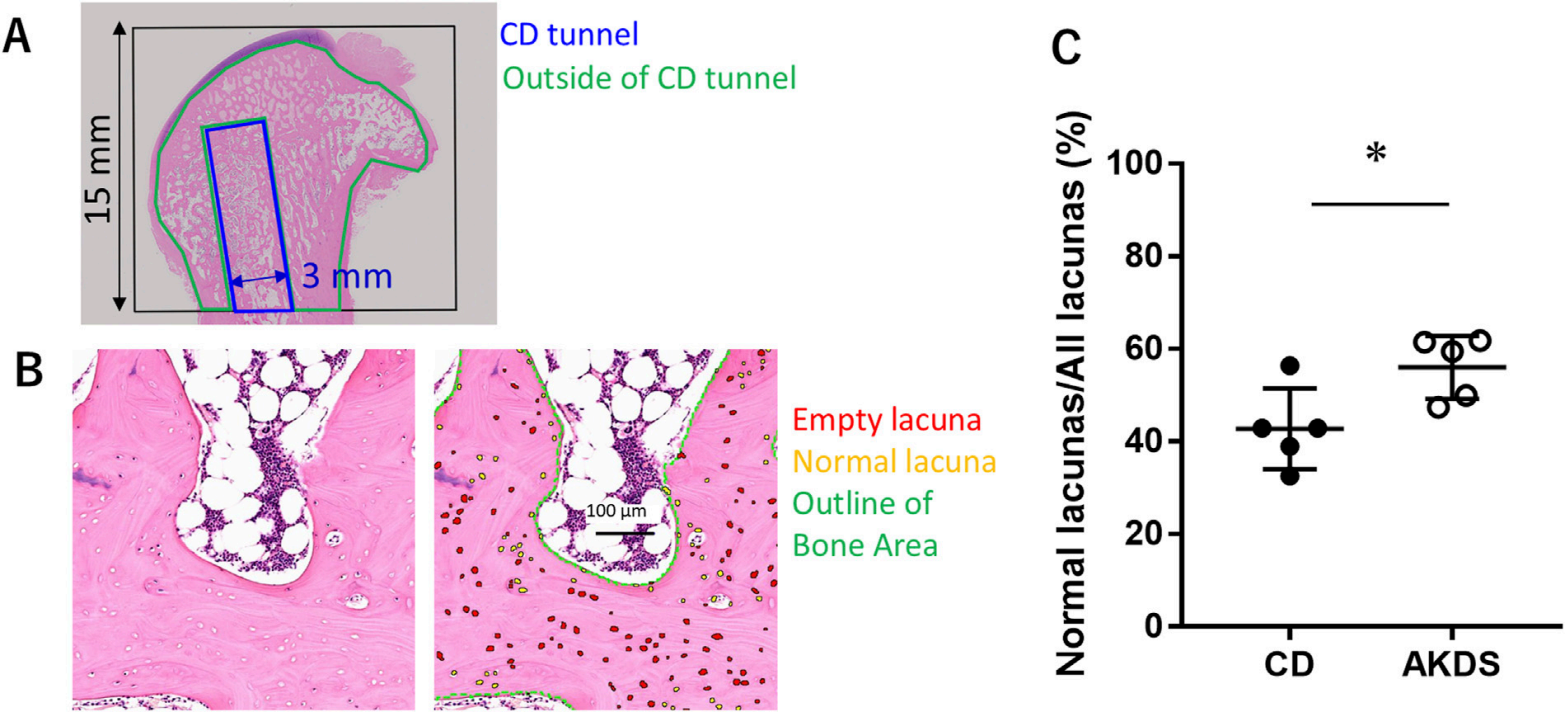
**(A)** The black box shows the region of interest for qualitative analysis. Blue indicates the CDT. Green indicates the area outside the CDT. **(B)** The left panel shows the bright field, and the right panel shows the same region as the right panel, with labels for empty lacunae (red), normal lacunae (yellow), and outlines of the bone area (green). **(C)** Results of qualitative histological analysis outside the CDT showing the ratio of normal lacunae to all lacunae. CD, core decompression; CDT, core decompression tunnel; AKDS, AKDS001 microspheres. *p < 0.05. Error bar: standard deviation.

In the histologic examination, all the animals in the CD-only group showed necrotic and fibrotic changes at 12 weeks after CD surgery (Figure 6). More viable bone tissue was present in the AKDS001 MS-treated groups than in the CD-only group (Figure 6A). Compared with those in the CD group, the median fibrosis scores were lower in the middle- and high-dose groups, and the median necrosis scores were lower in the AKDS001 MS-treated groups at all tested doses (Figures 6B,C). The difference in the necrosis score between the middle-dose group and the control group was statistically significant. However, the osteoblast score and osteoclast scores were low and similar in all the groups at this time point (Supplementary Table S6). The quantitative histological analysis revealed that the percentage of empty lacunae per total lacuna decreased outside the CD tunnels in the femoral head (Figure 7).

### 3.5 Safety of AKDS001 MS treatment in the ONFH dog model

In animals that received a higher dose (100 µg/site, 200 µg/body) of AKDS001 MS for safety assessment, increased trabecular bone and reduced fibrosis were observed around the injection sites, including the femoral head and neck, but no other treatment-related effects were observed. The plasma concentration of AKDS001 MS group was quite low even at maximum level and below the lower limit of quantification (BLQ, <10 pmol/L) at 33 days postdosing, while that of the CD group were all BLQ (Table 2). Ectopic bone formation in the intraarticular space was not observed in any of the animals. There were no abnormal changes observed in the clinical examinations, gait observations, body weights, food consumption, ophthalmic features, electrocardiographic features, hematological features, biochemical analysis, urinalysis, gross pathology or organ weights.

**TABLE 2:** Toxicokinetic parameters in ONFH dog.

Dose	C_max_ (pmol/L)	T_max_ (h)	AUC_0-t_ (pmol·h/L)
0 µg/site (CD only)	BLQ	BLQ	BLQ
100 µg/site	127.6 ± 36.2	5.0 ± 9.3	20436.8 ± 6,125.6

Abbreviations: C_max_, maximum concentration; T_max_, time to peak drug concentration; AUC, area under the curve; BLQ, below the lower limit of quantification. Data are presented as arithmetic mean ± standard deviation.

## 4 Discussion

There is growing evidence obtained from preclinical research that EP4 receptor agonists have therapeutic potential for osteoporosis and bone healing. However, their efficacy in ONFH models has not been investigated to date. In this study, the sustained local release of an EP4 receptor agonist, AKDS001, delivered through MSs, significantly enhanced bone regeneration in the necrotic bone tissue of the femoral head. The canine ONFH model employed in this study, induced by an ethanol injection into the femoral head, achieved a 100% incidence rate and accurately reproduced key early-stage pathological features of ONFH (Wang et al., 2013; Cui et al., 2021; Li et al., 2023). The necrotic lesions were well localized around the injection site, allowing for precise targeting of the affected area by core decompression. This anatomical characteristic enabled intraosseous drug administration *via* the same route as the CD procedure, thereby closely reflecting clinical application. Beagle dogs also offered an appropriate size and anatomy for surgical manipulation, making them a practical and translationally relevant model for evaluating intraosseous delivery of the clinical AKDS001 MS formulation. Furthermore, the use of the same species for both efficacy and toxicological assessments facilitated estimation of the therapeutic window, in alignment with international regulatory guidelines for non-rodent evaluation (Foster et al., 2022; International Conference on Harmonisation ICH, 2009).

In our study, the control group that underwent CD alone exhibited minimal bone regeneration at 12 weeks, which was indicated by a lower BMD in the CD tunnel than in the contralateral intact femurs, based on CT analysis. On the other hand, a previous study (Simank et al., 2001) using a sheep ONFH model induced by pure ethanol injection reported a higher BMD in and around the CD tunnel than in the contralateral intact femurs within 12 weeks after CD surgery. The slower bone repair observed in dogs than in sheep is inconsistent with known bone remodelling cycles, which are shorter in dogs than in sheep (Taguchi and Lopez, 2021). Nevertheless, the ethanol-induced ONFH in this canine model study was more severe and could not be fully repaired with CD alone. These findings suggest that the model is useful for evaluating combination drug treatments involving CD and drug candidates such as AKDS001.

AKDS001 MSs consistently increased new bone volume in CD tunnels in both intact and ONFH dog models. The bone regenerative effects of AKDS001 MSs appeared to be more pronounced in the animals with intact femurs than in ONFH model animals. However, even in the ONFH models, the AKDS001 MS-treated groups achieved a bone volume and BMD comparable to those of intact femurs following CD surgery. Histological assessments further revealed a greater presence of newly formed bone tissues with normal lacunae in the femoral heads of dogs treated with AKDS001 MSs and CD than in those treated with CD alone. Notably, this improvement extended to regions outside the CD tunnels, where necrotic tissue had not been removed by CD surgery. Typically, necrotic trabeculae in the AKDS001 MS-treated femurs were embedded in the newly formed bone. In a previous study, we reported that AKDS001 MSs increased osteoblastic activity and suppressed bone resorption, leading to increased new bone volume in human xenografts through minimodeling (Ukon et al., 2022). These mechanisms likely contributed to the promotion of bone formation even in areas where necrotic bone was not removed. Given that bone apposition and the gradual replacement of necrotic bone, known as creeping substitution, are key processes in ONFH repair (Cui et al., 2021; Fondi and Franchi, 2007), enhancing bone formation while suppressing bone resorption may represent an effective therapeutic strategy for ONFH. Kim et al. (Kim et al., 2014) reported that the local administration of bone morphogenetic protein (BMP)-2, a key regulator of bone formation, in combination with ibandronate resulted in promoted new bone formation and reduced bone resorption compared with the effects of BMP-2 alone during nonweight-bearing treatment in a pig model of ischemic osteonecrosis. However, heterotopic ossification was observed. Additionally, CD combined with the local administration of zoledronate and cell therapy has shown potential in delaying the progression of ONFH in the early and middle stages (Ma et al., 2021). These findings collectively suggest that promoting bone formation while concurrently reducing bone resorption is a promising therapeutic approach for bone regeneration in ONFH.

AKDS001 MSs, administered at a dose at least 10 times greater than the effective dose, were well tolerated with no systemic effects, including diarrhea, vomiting, injection site erythema, or abnormalities in biochemistry and hematology, which are reported in clinical trials of systemic EP4 agonists for ulcerative colitis or heart failure (Nakase et al., 2010; Ward et al., 2016). Localized injections appear to be critical for maximizing both the safety and efficacy of AKDS001 MSs, as this approach enables direct delivery of the drug formulation to regions with compromised blood supply due to necrosis (Aya-ay et al., 2007). In the present study, no heterotopic ossifications were observed following AKDS001 MS treatment, which is consistent with the findings of a previous study showing that another EP4 agonist did not induce ectopic bone formation under conditions in which BMP-2 did (Toyoda et al., 2005). In addition, the AKDS001 MSs did not induce osteophyte formation when it was intraarticularly injected into the hip joints, suggesting sufficient safety even in cases where the formulation may leak into the joint space. Collectively, these findings suggest that AKDS001 MS enhances the efficacy of CD by facilitating bone regeneration, even in necrotic regions, without any safety concerns.

This study has several limitations. First, no femoral head collapse was observed in the animal models used, which is consistent with previous studies using various ONFH models, except for bipedal species such as chickens and emus, which are less suitable for drug discovery and development studies (Li et al., 2023). Our previous clinical trial demonstrated that rhFGF-2-loaded gelatin hydrogels enhanced radiological bone regeneration and prolonged the joint preservation time. This finding supports the hypothesis that joint preservation can be achieved through bone regeneration in necrotic lesions. However, human clinical trials are necessary to confirm the efficacy of AKDS001 MSs in preventing or delaying collapse in early-stage ONFH patients. Second, this study did not compare the efficacy of AKDS001 MSs with that of other therapeutic agents, such as rhFGF-2, BMP-2, bone marrow concentrates, or MSCs. Although our results indicate that AKDS001 MSs are effective and safe for ONFH treatment, further studies with direct comparisons are needed to better define its relative advantages and therapeutic characteristics. Third, the observation period in this study is limited. While our findings suggest that AKDS001 MS treatment effectively promotes new bone formation within 3 months, which is a duration approximately twice as long as the drug’s release period, but this timeframe is still considered relatively short, and the longevity of its effects remains to be determined. Moreover, although histological analysis at 3 months post-dosing did not reveal significant changes in osteoblast or osteoclast numbers, it is possible that dynamic cellular or molecular responses may have occurred at earlier time points, particularly during the drug’s active release phase. Future studies incorporating both shorter and longer observation periods will be essential to better characterize the pharmacological profile and durability of AKDS001 MS effects. Finally, this study did not evaluate the effects of AKDS001 on angiogenesis. The PGE2–EP4 signaling pathway is known to promote angiogenesis by stimulating endothelial cell migration and tube formation (Rao et al., 2007; Zhang and Daaka, 2001), both of which are cellular mechanisms closely linked to bone formation and repair (Hankenson et al., 2011; Grosso et al., 2017). Therefore, activation of the EP4 receptor may enhance not only osteoblastic differentiation but also angiogenesis, contributing to increased bone regeneration. Assessing the impact of AKDS001 on angiogenic responses would help clarify the mechanisms underlying its bone-regenerative effects. Future studies employing techniques such as immunohistochemistry or micro-CT angiography would be valuable for this purpose.

## 5 Conclusion

Local administration of AKDS001 MSs combined with CD surgery significantly enhanced bone regeneration in necrotic lesions in the canine ONFH model, demonstrating both efficacy and favorable safety with local tolerability. Nevertheless, further studies are warranted to validate this therapeutic approach, particularly its efficacy in preventing femoral head collapse and improving clinical outcomes.

## Data Availability

The raw data supporting the conclusions of this article will be made available by the authors, without undue reservation.
