# Supplementary material for: EP4 receptor stimulation in combination with core decompression therapy enhanced bone regeneration in a canine model of osteonecrosis of femoral head
Source: Front Bioeng Biotechnol. 2025 Aug 14;13:1622918. doi: 10.3389/fbioe.2025.1622918 (PMC12391736; doi:10.3389/fbioe.2025.1622918)
Supplement: Supplementary file 1 [file DataSheet1.pdf]

Supplementary Material

1 Supplementary Figures and Tables

1.1 Supplementary Figures

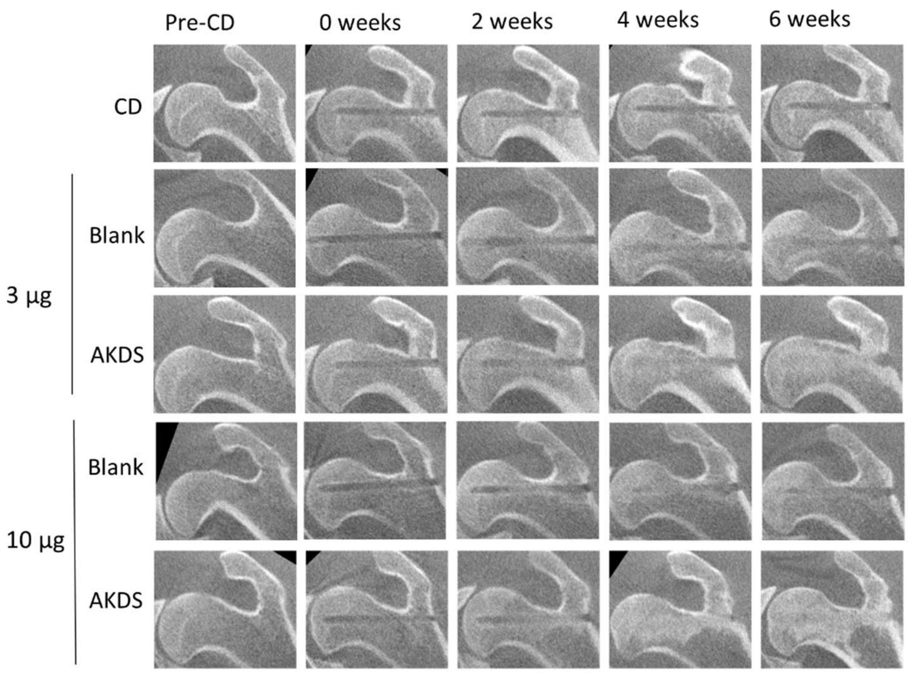

Supplementary Figure S1. Representative images of in vivo computed tomography after core decompression sugery for the dog femoral head. CD, core decompression; Blank, blank microspheres; AKDS, AKDS001 microspheres.

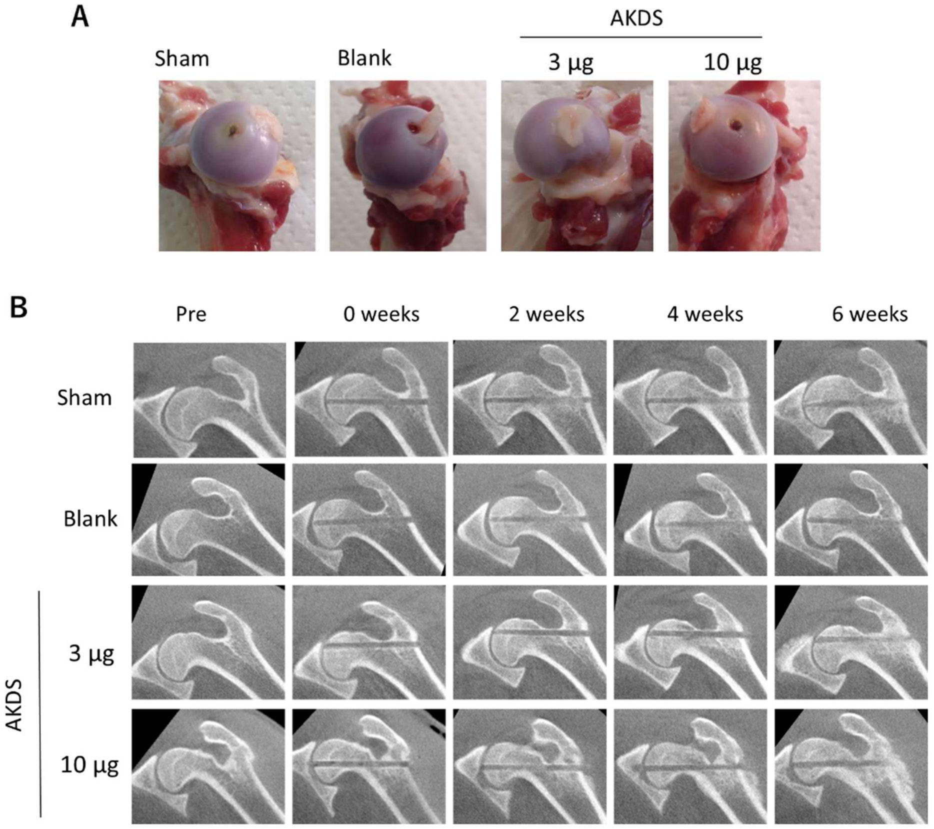

Supplementary Figure S2. (A) Photographs for gross observation of articular surface of femoral head at 6 weeks after intra-articular injection. (B) In vivo computed tomography images after intra-articular injection. Pre, pre-surgery; 0 weeks, immediately after injection; 2 weeks, 2 weeks after injection; 4 weeks, 4 weeks after injection; 6 weeks, 6 weeks after injection. Sham, drilling only; Blank, blank microspheres at the same volume as 10 µg; AKDS, AKDS001 microspheres.

1.2 Supplementary Tables

Animal No.	Treatment for Left Femur	Treatment for Right Femur
**1**	Blank MS (high dose)	CD only
**2**	AKDS001 MSs (3 µg)	Blank MSs (low dose)
**3**	AKDS001 MSs (10 µg)	Blank MSs (high dose)
**4**	Blank MS (low dose)	AKDS001 MSs (3 µg)
**5**	Blank MS (high dose)	AKDS001 MSs (10 µg)
**6**	CD only	Blank MS (high dose)
**7**	Blank MSs (high dose)	AKDS001 MSs (10 µg)
**8**	CD only	Blank MSs (high dose)
**9**	Blank MS (low dose)	AKDS001 MSs (3 µg)
**10**	AKDS001 MSs (10 µg)	Blank MSs (high dose)
**11**	AKDS001 MSs (3 µg)	Blank MS (low dose)
**12**	Blank MSs (high dose)	CD only

Supplementary Table S1. Animal allocation for CD surgery in intact femur dogs. CD, core decompression; MSs, microspheres.

**Osteoblasts**	**Score**	**Osteoclasts**	**Score**
>50% trabecular bone surface occupied by osteoblasts	4	>50% trabecular bone surface occupied by osteoclasts or scalloped edge	4
Approximately 25%–50% of trabecular bone surface occupied by osteoblasts	3	Approximately 25%–50% of trabecular bone surface occupied by osteoclasts or scalloped edge	3
Approximately 11%–25% of trabecular bone surface occupied by osteoblasts	2	Approximately 11%–25% of trabecular bone surface occupied by osteoclasts or scalloped edge	2
Approximately 1%–10% of trabecular bone surface occupied by osteoblasts	1	Approximately 1%–10% of trabecular bone surface occupied by osteoclasts or scalloped edge	1
None	0	None	0

Supplementary Table S2. Scoring criteria for histological assessments of osteoblasts and osteoclasts.

**Human MSCs**	**Age (years)**	**Sex**	**Race**	**AKDS001 EC_{50} (nM)**
Control	31	M	Black	11.4
	79	F	Caucasian	13.4
	66	F	Caucasian	13.0
Hip fracture	80	F	Asian	19.9
	82	F	Asian	20.0
	94	F	Asian	23.6
Osteonecrosis	70	F	Asian	20.6
	72	F	Asian	23.2
	27	M	Asian	19.7

Supplementary Table S3. Values of AKDS001 on cAMP production in human MSCs. EC_{50}, half-maximal effective concentration; cAMP, cyclic adenosine monophosphate; MSCs, mesenchymal stem cells; M, male; F, female.

Parameter	Side	CD	AKDS001		
			1	3	10
**BMD**	Left (treated)	0.20 ± 0.04	0.32 ± 0.07	0.35 ± 0.03	0.36 ± 0.08
**(gHA/cc)**	Right (intact)	0.20 ± 0.03	0.22 ± 0.03	0.20 ± 0.02	0.22 ± 0.05
**BV/TV**	Left (treated)	28.6 ± 7.9	52.6 ± 12.7	58.9 ± 5.4	59.9 ± 15.4
**(%)**	Right (intact)	31.6 ± 4.4	35.5 ± 5.4	32.3 ± 4.3	33.9 ± 7.3
**TMD**	Left (treated)	0.43 ± 0.04	0.51 ± 0.05	0.51 ± 0.04	0.51 ± 0.03
**(gHA/cc)**	Right (intact)	0.45 ± 0.03	0.47 ± 0.02	0.46 ± 0.02	0.47 ± 0.02

Supplemental Table S4. Results of micro-computed tomography analysis for ROI-1 in the dog ONFH model. Data are presented as mean ± standard deviation. CD, core decompression; AKDS001 MS, AKDS001 microspheres; BMD, bone mineral density; BV/TV, bone volume/total volume; TMD tissue mineral density.

Parameter	Side	CD	AKDS001		
			1	3	10
**BMD**	Left (treated)	0.27 ± 0.07	0.36 ± 0.13	0.36 ± 0.11	0.41 ± 0.11
**(gHA/cc)**	Right (intact)	0.34 ± 0.04	0.36 ± 0.03	0.35 ± 0.05	0.37 ± 0.04
**BV/TV**	Left (treated)	44.9 ± 12.9	60.9 ± 23.7	59.9 ± 23.6	70.4 ± 17.8
**(%)**	Right (intact)	61.2 ± 7.3	64.5 ± 6.1	62.7 ± 7.7	64.8 ± 6.8
**TMD**	Left (treated)	0.46 ± 0.04	0.50 ± 0.06	0.49 ± 0.06	0.52 ± 0.04
**(gHA/cc)**	Right (intact)	0.47 ± 0.03	0.49 ± 0.02	0.49 ± 0.03	0.50 ± 0.02

Supplementary Table S5. Results of micro-CT analysis for ROI-2 in the dog ONFH model. Data are presented as mean ± standard deviation. CD, core decompression; AKDS001 MS, AKDS001 microspheres.

Parameter	CD	AKDS001		
		1	3	10
Osteoblast scores	1 (0.13)	1 (0.22)	1 (0.34)	1 (0.11)
Osteoclast scores	0 (0.13)	0 (0.15)	0 (0.00)	0 (0.13)

Supplementary Table S6. Results of histological assessments of osteoblasts and osteoclasts. Data are presented as median (standard error). CD, core decompression; AKDS001 MS, AKDS001 microspheres; BMD, bone mineral density; BV/TV, bone volume/total volume; TMD tissue mineral density.
